# Gene therapy for ADA‐SCID, the first marketing approval of an *ex vivo* gene therapy in Europe: paving the road for the next generation of advanced therapy medicinal products

**DOI:** 10.15252/emmm.201707573

**Published:** 2017-04-10

**Authors:** Alessandro Aiuti, Maria Grazia Roncarolo, Luigi Naldini

**Affiliations:** ^1^ San Raffaele Telethon Institute for Gene Therapy (SR‐Tiget)IRCCS San Raffaele Scientific InstituteMilanItaly; ^2^ Vita Salute San Raffaele UniversityMilanItaly; ^3^ Present address: Division of Stem Cell Transplantation and Regenerative MedicineDepartment of PediatricsISCBRMStanford UniversityStanfordCAUSA

**Keywords:** Genetics, Gene Therapy & Genetic Disease

## Abstract

Gene and cell therapy research recently reached a fundamental milestone toward the goal to deliver new medicines for orphan diseases. In 2016, the European Commission granted market approval to GlaxoSmithKline (GSK) for *ex vivo* hematopoietic stem cell (HSC) gene therapy for the treatment of adenosine deaminase (ADA)‐deficient severe combined immunodeficiency (SCID), a very rare congenital disorder of the immune system. The new medicine, named Strimvelis™, is an advanced therapy medicinal product (ATMP) (Salmikangas *et al*, 2015) originally developed by the San Raffaele Telethon Institute for Gene Therapy (SR‐Tiget), a joint venture between Telethon Foundation and San Raffaele Scientific Institute. This ATMP is the first *ex vivo* stem cell gene therapy to receive regulatory approval anywhere in the world. Strimvelis™ consists of a single infusion of autologous gene‐corrected HSC and is prepared from the patient's own bone marrow (BM) HSCs, which are genetically modified using a gamma‐retroviral vector to insert a functional copy of the ADA gene.

ADA‐SCID is a life‐threatening disease, which is typically fatal within the child's first years of life, because of lymphopenia, failure to thrive, and recurrent and opportunistic infections (Gaspar *et al*, 2009). Non‐immunological features include skeletal abnormalities, neurological deficits, and hepatic dysfunction. Although a BM transplant from a human leukocyte antigen (HLA)‐matched related donor is recommended as first‐line treatment, this is only available for a minority of patients (< 25%) and survival after transplantation falls significantly as HLA matching decreases, ranging from 86% to 43%, albeit improving in most recent years (Hassan *et al*, 2012). In the absence of an HLA‐matched donor, patients can be treated with weekly intramuscular injections of enzyme replacement therapy (ERT) consisting of bovine ADA. However, the efficacy of long‐term ERT tends to decrease over time, and it does not prevent chronic complications and can amount to staggeringly high costs (Gaspar *et al*, 2009).

Approval of gene therapy for ADA‐SCID arrives 25 years after the first gene therapy attempt in humans. ADA‐SCID was considered an ideal candidate for somatic cell gene therapy because of the ubiquitous expression of the ADA enzyme in normal conditions and the survival advantage of ADA‐expressing cells (Ferrari *et al*, 1991). The pioneering work of research groups in Italy and the USA provided fundamental proof‐of‐concept that gene therapy was feasible and had an acceptable safety profile. The first approaches were based on multiple infusions of peripheral blood lymphocytes transduced with a gamma‐retroviral vector, which resulted in sustained engraftment of ADA‐expressing T cells (Blaese *et al*, 1995; Bordignon *et al*, 1995) (Fig 1). A parallel strategy was developed aimed at infusing BM or umbilical cord blood progenitors to patients without any preparative conditioning (Bordignon *et al*, 1995; Kohn *et al*, 1995). These studies resulted in low vector marking of progenitor‐derived cells with insufficient ADA production, and patients remained on ERT.

**Figure 1:**
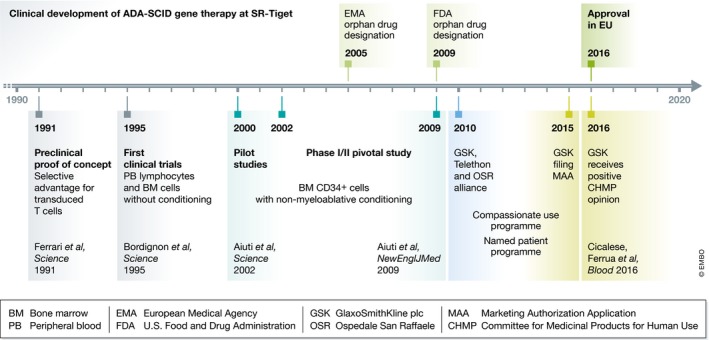
Schematic representation of the key scientific and regulatory milestones in the clinical development of ADA‐SCID gene therapy, leading to its approval in the EU

A breakthrough was achieved when two successful pilot studies conducted at Hadassah Hospital (Jerusalem, Israel) and SR‐Tiget introduced an improved gene transfer protocol for BM hematopoietic stem and progenitor cells and the use of non‐myeloablative chemotherapy regimen with busulfan in advance of gene therapy to make space for the transduced progenitors in the BM (Aiuti *et al*, 2002). Since none of the patients received concurrent ERT, the efficacy of gene therapy as single treatment could be fully assessed, exploiting at the same time the growth advantage for ADA‐transduced cells. Following these initial studies, results were confirmed and extended in a phase I/II pivotal study conducted in 12 more patients at SR‐Tiget (Fig 1). Gene therapy resulted in sustained lymphoid reconstitution with gene‐corrected T cells, improvement of immune functions, and effective metabolic detoxification, in the absence of adverse events related to gene therapy (Aiuti *et al*, 2009).

Market approval of Strimvelis™ in the European Union (EU) was based on data collected from a total of 18 ADA‐SCID children treated from 2000 to 2011, with a median follow‐up of about 7 years (Cicalese *et al*, 2016). Survival was 100% and the majority of patients showed evidence of long‐term gene correction in T lymphocytes, sustained increase in lymphocyte counts, maintenance of robust immune reconstitution, significantly fewer severe infections over time, and continued physical growth. Whereas gene marking was high in the T‐cell compartment (averaging 70% at 1‐year follow‐up), in agreement with the survival advantage of gene‐corrected lymphoid cells, the level of gene marking was much lower in the myeloid compartment (median 1–2% in CD34^+^ cells), indicating limited transduction and/or engraftment of transduced HSC. Importantly, gene‐modified cells were detectable in multiple hematopoietic lineages and stable engraftment persisted throughout long‐term follow‐up in the majority of patients, indicating that, although at low levels, correction of multipotent stem cells was achieved.

Overall, the safety findings were in line with those expected in an ADA‐SCID population receiving chemotherapy with busulfan and undergoing immune reconstitution. Importantly, unlike these trials, no events indicative of leukemic transformation of transduced cell clones were reported. This positive outcome and the key role of conditioning were also reported by subsequent gene therapy studies for ADA‐SCID performed at other centers using different gamma‐retroviral vectors (Gaspar *et al*, 2011; Candotti *et al*, 2012).

The approval of ADA‐SCID gene therapy is the result of a joint effort among different stakeholders and exemplifies how the open cooperation between academic institutions, not‐for‐profit funding agencies, and pharmaceutical industry can overcome the many hurdles in developing new drugs for rare conditions.

Throughout the past 15 years, key research has been carried out aimed at studying the pathogenesis of immune and non‐immune manifestations of ADA‐SCID, optimizing gene transfer into HSC and investigating their *in vivo* behavior after engraftment (Aiuti *et al*, 2007). SR‐Tiget established dedicated infrastructures to support high‐quality preclinical studies and proof‐of‐concept ATMP clinical trials, including (i) a Good Laboratory Practice (GLP) test facility for preclinical studies (Carriglio *et al*, 2017), (ii) a Pediatric Clinical Research Unit with a multidisciplinary clinical team and a specialized clinical trial office, and (iii) a vector integration Core Unit to perform genome‐wide profiling of vector integration sites in preclinical and patient samples. However, when the medicinal product obtained orphan drug designation by the European Medicines Agency (EMA), the Telethon Foundation still faced the challenge of finding the resources to complete the drug development process to actually fulfill its mission to make the treatment available to all patients in need (Monaco & Faccio, 2017). The agreement signed by the Telethon Foundation, the San Raffaele Hospital, and GSK in 2010 provided the economic resources, the expertise, and the infrastructures required to complete clinical development, establish pharmaceutical production, and prepare for the launch of a new medicinal product.

Within this collaboration, the biotech company MolMed S.p.A., which had been involved since the beginning in producing vector and cells under Good Manufacturing Practices (GMP), applied its expertise in product development to optimize and standardize the manufacturing process achieving robustness and suitability for commercial supply.

The interactions with local ethics committees, regulatory authorities at the national (Italian Medicine Agency (AIFA) and Istituto Superiore di Sanità) and European levels (EMA committees and working parties) have been constructive throughout the process. Protocol assistance and pre‐submission meetings with these agencies provided effective advice on manufacturing and preclinical and clinical development. Despite the complexity and the many challenges faced by such a highly innovative product, the EMA Committee for Human Medicinal Products Use (CHMP)'s positive opinion was received within 10 months from the validated submission, and EU marketing was approved by the EU Commission within 12 months. AIFA approved Strimvelis™ pricing and reimbursement in Italy in < 2 months through an accelerated procedure. Similar agreements are under discussion in other EU countries.

Strimvelis™ is available for ADA‐SCID patients without a suitable HLA‐matched related donor and administered at present only at the San Raffaele Hospital in Milan due to the short current product shelf life and requirements for expertise in gene therapy and HSC transplantation. Indeed, *ex vivo* HSC gene therapy must comply with high standards in product management and supply chain, from the procurement of the autologous BM HSC to the medicine infusion. A robust quality system certified by the Joint Accreditation Committee (JACIE) has been established at the San Raffaele Stem Cell Program.

Patients treated with the experimental and licensed product will continue to be monitored since the long‐term effects of gene therapy (> 10–15 years) are not known. A long‐term prospective observational study is being implemented by GSK in accord with EMA recommendations to monitor the long‐term risks of insertional mutagenesis, oncogenesis, immunogenicity, and hepatic toxicity as well as the persistence of efficacy of ADA‐SCID gene therapy.

As of end of 2015, only six ATMPs were authorized in the EU since the implementation of ATMP regulations (1394/2007). Of these, only one has been reported to be actually administered as a commercial product, while the others have been discontinued or are still struggling to secure reimbursement. The approval of two new ATMPs in 2016 represents a turning point for the field, along with the extraordinary number of agreements between industrial biotech and academia that promise to reshape the field and increase our expectations on its potential impact on human health. The innovative legislative and regulatory pathways recently implemented in the EU (such as the Innovation Task Force, Priority Medicine (PRIME) initiative, Adaptive Pathway, and Accelerated Review), in the USA (Breakthrough Therapy Designation and Priority Review), and in Japan are expected to accelerate the development of ATMPs and help to bring safe and effective products to the market.

Although Strimvelis™ represents the first generation of gene therapy vectors for stem cell gene therapy, its safety and efficacy track record charts a clear path for ATMP development from early clinical experimentation to drug registration and marketing. The valuable outcomes of this process reach well beyond the benefit of ADA‐SCID patients and include the strategies developed to support the quality assessment, specifications and manufacturing of the ATMP, as well as its administration at the hospital bed, and finally, but not less importantly, the policies adopted for establishing its cost and reimbursement policy. These advances pave the way to further clinical development of newer generations of vectors with higher efficiency and safety of gene transfer, such as lentiviral vectors. Based on the current safety and efficacy data from clinical studies using the lentiviral vector platform, we expect a rapid growth of the applications of stem cell gene therapy in several inherited diseases and in cancer. Marketing authorizations for new ATMPs have already been requested in the EU or are likely to be filed within the next 2–3 years. Once approved, the next challenge will be to make these therapies accessible to patients worldwide. Pharmaceutical industries will be confronted with the necessity to standardize process development across different countries, guarantee adequate manufacturing capacity for the growing needs of expanded applications, establish a network of qualified treatment centers for administration of the new ATMPs, ensure long‐term monitoring and pharmacovigilance, and maintain sustainability and cost‐effectiveness of these personalized therapies. The successful collaboration between academia and industry established for ADA‐SCID gene therapy could represent a model to follow and to further expand upon.

## Conflict of interest

AA is the principal investigator of the ADA‐SCID gene therapy trial sponsored by GSK. GSK acquired the license from Telethon and Ospedale San Raffaele, which are entitled to receive milestone payments and royalties upon commercialization of this and other gene therapies for genetic diseases.
